# Size but not relatedness drives the spatial distribution of males within an urban population of *Anolis carolinensis* lizards

**DOI:** 10.1002/ece3.7248

**Published:** 2021-02-14

**Authors:** William David Weber, Nicola M. Anthony, Simon P. Lailvaux

**Affiliations:** ^1^ Department of Biological Sciences University of New Orleans, New Orleans LA USA; ^2^ Department of Biology University of Maryland College Park MD USA

**Keywords:** *Anolis carolinensis*, pedigree, relatedness, spatial distribution, territoriality

## Abstract

The way that individuals are spatially organized in their environment is a fundamental population characteristic affecting social structure, mating system, and reproductive ecology. However, for many small or cryptic species, the factors driving the spatial distribution of individuals within a population are poorly understood and difficult to quantify. We combined microsatellite data, remote sensing, and mark–recapture techniques to test the relative importance of body size and relatedness in determining the spatial distribution of male *Anolis carolinensis* individuals within a focal population over a five‐year period. We found that males maintain smaller home ranges than females. We found no relationship between male body size and home range size, nor any substantial impact of relatedness on the geographic proximity. Instead, the main driver of male spatial distribution in this population was differences in body size. We also found no evidence for offspring inheritance of their parent's territories. Males were never sampled within their father's territory providing strong support for male‐biased dispersal. This study introduces a novel approach by combining standard mark release capture data with measures of pairwise relatedness, body size, and GPS locations to better understand the factors that drive the spatial distribution of individuals within a population.

## Graphical abstract

We have examined an isolated population of *Anolis carolinensis* over a five‐year period in order to answer the question of how individuals in that population positioned themselves from one another. Using combined methodology, our study shows that females *A. carolinensis* hold larger home ranges than males and that a male intrasexual size dimorphism may be the primary driver of how males position themselves.

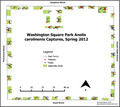

## 1 INTRODUCTION

The way that animals distance themselves from one another within their preferred habitats has important consequences for both the reproductive ecology and social structure of a species. Age, sex, size, and mating system can all affect the spatial distribution of individuals, as can seasonal effects on food abundance or nesting site quality *(*Clausen et al., 1998; Kesler & Haig, 2007; Seymour et al., 2003). Individual movements within a home range, territoriality, dispersal/philopatry, and interactions among relatives further affect how individuals are arranged with respect to each other in their habitat. Fine‐grained studies of individual movements within populations have revealed novel insights into the behavioral ecology of a range of vertebrate taxa (Hartman et al., 2016; Stradiotto et al., 2009; Winck et al., 2011). For example, such studies have uncovered previously unknown harem mating systems in the hawkfish *Paracirrhites forsteri* (Kadota & Sakai, 2016). However, few studies have examined the relative importance of different competing biotic and abiotic factors in driving the spatial organization of individuals within a population.

One common driver hypothesized to influence the distribution of individuals within a population is intraspecific variation in body size. Territoriality and home range size are positively, but not exclusively, correlated with body size in many animals (Campioni et al., 2013; Escudero et al., 2020; Haenel et al., 2003; Ramos et al., 2015). In sexually dimorphic species, males will often maintain larger home ranges than females (Baber & Coblentz, 1986; Cranford, 1977; Kitchings & Story, 1984; Perry & Garland, 2002) and may demonstrate territoriality by defending a smaller part of that home range against other males (Subrahmanyam & Sambamurty, 2006). Territoriality drives aggressive interactions between males and in many cases leads to the dispersal of male offspring (Alonso & Alonso, 1992). By contrast, females tend to be philopatric, remaining in the same areas throughout their lives for reasons ranging from familiarity with and access to parental resources, to avoidance of outbreeding or the costs of dispersal (Pusey, 1987). In general, males exhibit higher territoriality, aggression, and dispersal, whereas philopatry tends to be a female trait (Parish et al., 2006).

One of the potential consequences of philopatry is the future inheritance of a territory and its concomitant fitness benefits from a dominant parent (Buston, 2004; Ragsdale, 1999). Territorial inheritance is often considered within the context of group living species or cooperative breeders (Desjardins et al., 2008) and can result from either female inheritance and male dispersal (e.g., Holekamp & Sherman, 1989) or male inheritance and female dispersal (e.g., Komdeur & Edelaar, 2001). However, even in species where offspring remain in their natal range and do not assist with breeding, those individuals that are larger, older, or more dominant stand to benefit through inheritance of a parental territory, whereas others, for whom the costs of acquiring or maintaining that territory are too high, may instead gain greater benefits through dispersal (Kokko & Ekman, 2002). Although body size, sex‐biased dispersal, and patterns of relatedness are key aspects of both the spatial distribution of individuals within their habitats and determinants of their overall mating success, quantifying such processes in small and/or cryptic species that lack a well‐defined social structure remains a challenge.

Offspring dispersal affects the genetic structure of a population (Wright, 1946) which in turn impacts gene flow (Slatkin, 1987). Population density within a habitat also influences population characteristics such as disease transfer rates (Hales et al., 2002) and inbreeding depression (Huisman et al., 2016). Dispersal of an individual from its natal range will therefore alter the degree of genetic differentiation (and conversely the level of relatedness) expected between individuals. Evidence for an effect of geographic distance on pairwise relatedness between individuals can be indirectly quantified through tests of isolation by distance (IBD), whereby the relatedness of individuals is inversely proportional to the distance between them (Loiselle et al., 1995). However, there may not be any clear IBD relationship if close relatives do not disperse or if juvenile dispersal is very high. Furthermore, social familiarity may also influence the spatial distribution of individuals in their habitats. For example, territorial males (Jaeger, 1981; Lancaster & Jaeger, 1995; Qualls & Jaeger, 1991) are often more tolerant of their neighbors than nomadic individuals, regardless of their relatedness (i.e., the Dear Enemy Hypothesis [Fisher, 1954]). Nonetheless, combining IBD with long‐term, fine‐scaled observations of territory ownership and habitat use across multiple generations could potentially shed light on the drivers of habitat use, territory ownership, and, ultimately, spatial distribution of individuals within their habitats.

The green anole lizard (*Anolis carolinensis*) has long been established as a model system in ecology and evolution, yet relatively little is known regarding the factors influencing the spatial distribution of individuals within populations of this species. Green anoles exhibit a polygynous, female defense mating system whereby females establish a home range and defend small, resource‐based territories while males defend a territory containing multiple females (Jenssen et al., 2001; Jenssen & Nunez, 1998; Ruby, 1984) leading to a high degree of male territoriality and accompanying high frequency of male–male combat (Lailvaux & Irschick, 2007; Orrell et al., 2004). A recent study by Kamath and Losos (2017 & 2018) questioned the phenomenon of territoriality in *Anolis* in general; however, that study has in turn received criticism (Bush & Simberloff, 2018; Stamps, 2018), and there is clear empirical evidence that the green anole mating system in particular is dominated by male territoriality and combat (e.g., Jenssen et al., 1995, 2001; Jenssen & Nunez, 1998; McMillan & Irschick, 2010; Nunez et al., 1997). Jenssen et al., (1995) showed that adult males maintain the same territory throughout the breeding season, with incursions and takeovers appearing to be rare. While male territories do not overlap, females traverse the territories of different males.

Previous studies of green anole populations in the New Orleans area have documented the presence of an intraspecific dimorphism based on male head morphology and bite force. Males with a snout–vent length less than 64 mm are considered “lightweights,” whereas those of 64 mm or more are considered “heavyweights” (Husak et al., 2009; Lailvaux et al., 2004; Vanhooydonck et al., 2005). Heavyweight males have relatively larger heads and bite forces for their size and are clearly distinguishable based on head morphology alone. This intraspecific dimorphism in adult male size also indicates that territory ownership in nature might be skewed heavily toward larger and competitively superior heavyweight males; indeed, previous studies have suggested that smaller lightweights are excluded from holding prime territories until they become large enough to compete with resident heavyweights (Irschick & Lailvaux, 2006; Lailvaux et al., 2004). This further suggests that smaller males should cede territories to larger males and disperse to an area either to establish their own territory or acquire mates by employing a sneaker strategy (Orrell & Jenssen, 2003). Previous studies in the Caribbean lizard *Anolis roquet* have shown evidence for male‐biased dispersal (Johansson et al., 2008). In contrast, studies on the brown anole *Anolis sagrei* indicate that while both sexes are highly philopatric, male dispersal is dependent on body size whereas female dispersal is more likely to be dependent on resource availability (Calsbeek, 2009). However, no studies to date have examined the effect of either relatedness or body size on home range behavior and inheritance in the green anole.

In the present study, we tested the influence of both variables (body size and relatedness) on the spatial distribution of males within an urban population of green anoles in Washington Square Park, New Orleans. By marking and genotyping every captured individual over a five‐year period, we built a partial pedigree and used locations of capture sites to construct spatial distribution maps of individuals in the population for each sampling season. We tested four hypotheses and associated predictions regarding the spatial distribution of individuals at this site: (a) Home range sizes differ significantly between males and females. Specifically, we predict that home range size will be greater for males than females. (b) Home range size is correlated with body size. We predict that the largest males will have the largest home ranges. (c). Male–male spatial distribution is driven by body size. Here, we predict that geographic distance between males is positively correlated with differences in body size. (d) Dispersal is sex‐biased. We predict that male offspring will disperse from natal home ranges more than female offspring. We further predict that, because of this male‐biased dispersal, females will inherit territories from their mothers, but males will not inherit territories from their fathers.

## 2 METHODS

### 2.1 Study site

We conducted this study on a population of free‐ranging green anole lizards in Washington Square Park (N29.965005°, W90.057302°), located in New Orleans, Louisiana, USA. The park is one hectare in size and is surrounded by an iron fence, the exterior of which is bordered by a concrete side‐walk adjacent to the roads that entirely delimit the park. Aside from the area within the park, there is little ideal habitat that would maintain a population outside of the park making dispersal from the park, while likely, rare. Green anole habitat primarily comprises bushes of the common cast‐iron plant (*Aspidistra elatior*) which fringe the interior of the park fence. These bushes stretch on average two meters into the park and represent the preferred habitat of green anoles within the city of New Orleans (Irschick et al., 2005). The interior of the park consists of open lawn and live oak trees (*Quercus virginiana*) found along the edges of the park. Although green anoles are observed on the trunks of the oak trees, the fences and cast‐iron plant bushes serve as primary habitat in this population, as this is where the vast majority of lizards were captured, and because previous studies have shown that individuals were not observed high on tree trunks or inhabiting the canopy (Irschick et al., 2005).

### 2.2 Animal mark–release–recapture

We sampled the green anole population of Washington Square Park (see supplemental image S1) in the spring (mid‐April to early May) and the fall (mid‐September to early October) of each year from 2010 until 2014. We captured lizards either by lassoing them with dental floss or by cupping them in hand, marked (with tape) each specimen's location in the bush, and gave it a unique identification number. After GPS coordinates of the capture site were recorded, we transported lizards to the laboratory at the University of New Orleans where they were permanently marked by a unique identification mark with injected visual implant elastomer tag (Northwest Marine Technology, Inc.) (Losos, 2009). We removed a tail tip of no more than 10mm from each individual with sterilized scissors and placed it into a vial of 95% ethanol. We sexed the lizards and then weighed them to the nearest 0.01g, with a Type XS107 Mettler‐Toledo scale (Mettler‐Toledo, LLC). We measured snout–vent length (SVL) to the nearest 0.01 mm with Rok digital calipers (Rok International Industry Co., Limited). Before their release, individuals were marked with a fade and water‐resistant marker just above the dorsal tail‐base to facilitate visual identification on subsequent collection days within the same season and to prevent recapture within the same sampling period. The marking was eliminated on the individual's next molting. Finally, we released lizards the morning after collection at the exact point of capture as indicated by the tape marker placed at the capture location. All methods were approved under IACUC protocol (UNO‐11‐004).

### 2.3 Home range assessment

**FIGURE 1:**
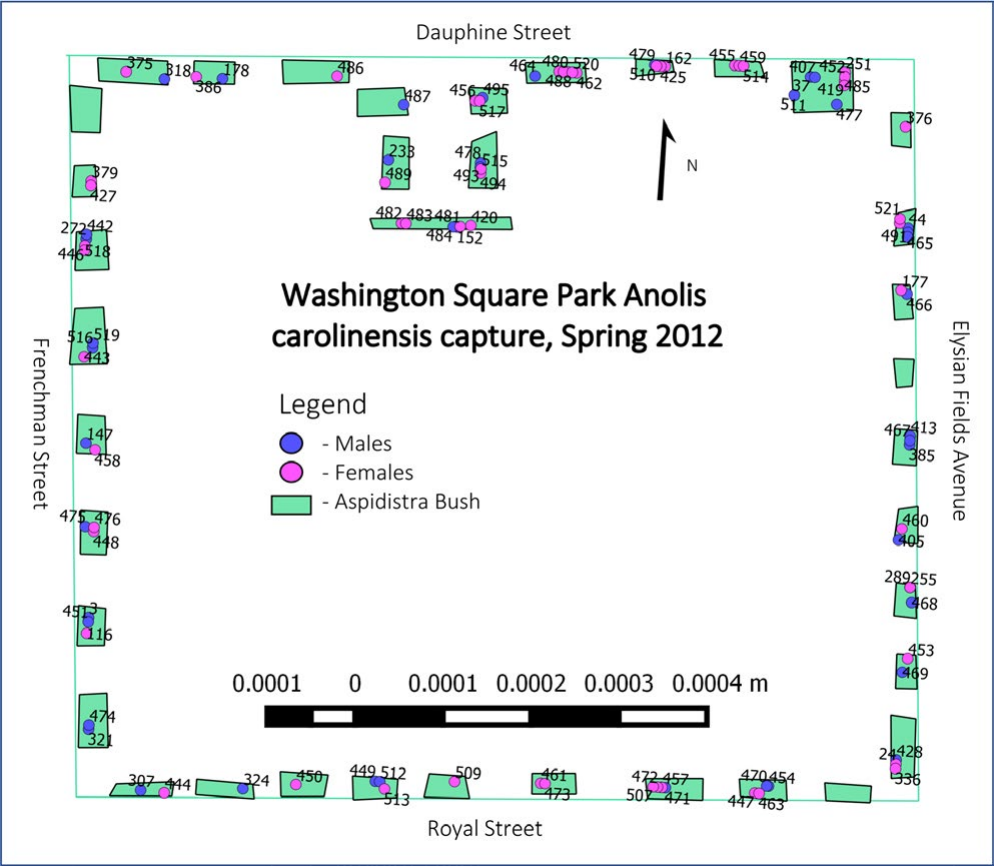
Study Site Maps map of Washington Square Park, New Orleans, LA, USA. This example of a sampling map from the spring of 2012 shows the “capture location” layers added atop habitats, blue dots indicated the capture location of a male and red dots a female. All dots are labeled with the individual's identification. Additional cohort maps can be viewed in supplemental material

We first constructed maps of individual capture locations using QGIS v 2.4.0.0 (QGIS Development Team, 2015) and a “Google Maps” overlay as the template for park boundaries, defined as the location of the perimeter fence. After measuring the length and width of each cast‐iron bush, we represented individual bushes as polygon shape files that were each assigned a number from 1 to 38 (Figure 1). This general habitat map served as a base map for all other analyses. Using the GPS coordinates obtained from each individual's capture location, we created a vector file for each cohort and overlaid those vectors on the base map. Cohorts could then be assigned locations and sorted by sex. We also incorporated the size of each male in the cohort vectors, so as to be able to segregate individuals according to their size (Figure 1; see also Figure S1). The limited accuracy of our GPS device (a device with an error rate of 0.08% equates to ±1.30 m) meant that we were only able to assign the location of an individual to the bush where captured.

The capture location of each individual for our purposes is therefore the geographic center of the bush where it was captured. In cases where individuals were captured as part of more than one cohort, multiple capture locations were compiled into another vector that displayed all the locations where those individuals were captured on the same map. Maps of individual capture locations were then used to assess home range. Because individuals rarely occur in the middle of the park, most likely due to the lack of suitable habitat, we used only the bushes and fence as dispersal routes. Due to the low number of recaptures (Rose, 1982), we created polygons of the area between an individual's farthest points of capture (cf. Lance et al., 2011). In short, when one individual is captured in one season, that capture location represents one data point, when captured in subsequent seasons, those locations become additional datapoints. The two datapoints farthest from one another are assumed to be the distal limits of the home range. We then created a polygonal area using the “Measure Line” tool in QGIS which included the distal data points, and all area in between (see Figure S3). QGIS then calculated the area within that polygon to give us an estimated individual's home range. We chose to use the method employed by Lance et al., (2011) because other models—for example that of Jennrich and Turner (1969)—will tend to overestimate actual home ranges (Stone & Baird, 2011). Home range data were normalized by log transformation. We then correlated home range size with an individual's SVL using the “cor.test” command in R (Version 3.3.3; R Core Team [2017]).

### 2.4 Spatial distribution of heavyweight versus lightweight males

We quantified spatial distribution of individuals in the park using the QGIS “Measure Line” tool and measured the geographic center of each bush to geographic center of every other bush in the habitat, creating a matrix of distances. As green anoles have not been observed in the center of the park, this area was not considered to be corridor of dispersal. The average geographic distance of each individual male from all other individual males within its cohort was then calculated using the average distance formula:

$$\overset{\prime }{d}=\frac{\varepsilon \left(d_{1}+d_{2}+d_{3}+\ldots \right)}{n‐1}$$

As with home range assessment, due to the limited accuracy of our GPS device, individual locations were assigned to a bush habitat, not to specific locations within the bush.

Once average geographic distances for all individuals were compiled, we tested for significant differences in this measure between heavyweight and lightweight males by conducting general linear models on the whole data set in R and controlled for male density in a given cohort by treating it as a covariate with body size. We performed this model on the whole male dataset and then separately with only heavyweights and again with only lightweights. We also tested average distance within each cohort using a pairwise Wilcox test corrected for multiple hypothesis testing.

### 2.5 Microsatellite genotyping and pedigree construction

We extracted genomic DNA from tail tips using the QIAGEN DNeasy Blood and Tissue extraction kit (QIAGEN, CA) using the manufacturer's protocol. Genotyping was conducted using eight highly polymorphic microsatellite loci located across five autosomal chromosomes and assembled into two multiplex assemblies of four loci each. Each reaction contained primers from previously published loci (Wordley et al., 2011) labeled with a fluorophore tag on the 5′ end (Table S1). We carried out both multiplex PCRs in a total volume of 10 μl using 5 µl 2X Multiplex PCR Kit (QIAGEN), 0.01 µl of each forward and reverse primer at a concentration of 1mg/ml, and 1µl of DNA [4–7 ng/µl] using the following conditions: Step 1, an initial denaturation at 95°C for 15 min followed by 35 cycles of step 2. Step 2, 94°C for 30 s, primer annealing at 55°C for 90 s, and an extension at 72°C for 60 s followed by a final 60°C extension period for 30 min. Microsatellite genotyping was carried out using an ABI 3100 Genetic Analyzer (Applied Biosystems) with the ROX‐500 size standard (GeneScan). Next, we visually inspected electropherograms using GENEIOUS (Biomatters) and binned the genotypes with FLEXIBIN (Amos et al., 2007). We tested all loci for deviations from Hardy–Weinberg and linkage equilibrium using ARLEQUIN, applying a Holm's‐Bonferroni sequential correction (Rice, 1989) with an initial alpha value of 0.0065 (Excoffier & Lischer, 2010). We also tested for the presence of null alleles, short allele bias, and the effects of stutter using MICROCHECKER v. 2.2.3 (Van Oosterhout et al., 2004).

For pedigree construction, we used the software program COLONY (Wang, 2013). We set mating system parameters for male and female polygamy, in a dioecious, diploid population, with the possibility for inbreeding. The run was set for “long” with a full‐likelihood analysis method set at medium precision, and no sib‐ship priors. Because we were unable to assess parentage through observation, the data set of 846 unique individuals was broken down into seasonal cohorts. For each cohort, we assumed all individuals to be potential offspring, all males as potential fathers, and all females as potential mothers. We then combined cohorts in a sequential, stepwise manner in which all the previous seasons’ cohorts were added to the succeeding cohort so that the parentage of all the previous cohorts served as the known paternity and maternity priors of the next. This procedure was conducted until we constructed an entire pedigree of all cohorts and individuals. The probability of a parent being included in the candidate genotypes was set at 50%, and a genotyping error rate of 1% was used in all constructions. The same analysis was run in triplicate and only parent–offspring relationships recovered in all three replicate runs with a *p*‐value less than 0.05 were retained for further analysis (Hoffman & Amos, 2005; Jones & Wang, 2010).

### 2.6 Relatedness analysis

We used the program SPAGeDi to estimate pairwise relatedness coefficients between all male–male dyads and to assess patterns of relatedness as a function of distance (Hardy & Vekemans, 2002; Loiselle et al., 1995). We calculated pairwise relatedness values between each heavyweight male and all other males of its cohort, and then to males captured within a given male's home range within a given cohort. A regression analysis was used to assess the relationship between the average pairwise relatedness of each male to all others in a given cohort with (a) the average geographic distance it was captured from all other males of that cohort, and (b) the average body size difference between it and all other males within a given cohort.

We then constructed spatial auto‐correlograms using the output from SPAGeDi and the five distance classes it produced in each cohort to determine at what spatial scale IBD was evident. Spatial auto‐correlograms were plotted in the Microsoft Excel 2010. Lastly, we also conducted Mantel tests to assess evidence for overall IBD within cohorts using the “ape” package in R (Paradis & Schlieb, 2018).

### 2.7 Home range inheritance

After parentage was assigned to our dataset (via the Colony pedigree), we then examined the capture locations of those offspring, to determine whether they had ever been captured within a parent's home range. Then, for each parent, we calculated the percentage of its offspring captured within and outside of its home range. Additional, with offspring presence/absence count data, we performed a Pearson's chi‐square test (with Yate's continuity correction) to ensure our findings were not being driven by randomness. This approach allowed us to assess whether offspring were philopatric to the natal home range and also whether they were dispersing.

## 3 RESULTS

### 3.1 Home range assessment

**FIGURE 2:**
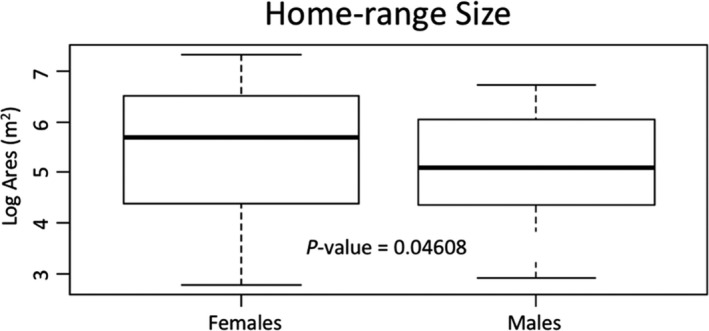
Home range area of green anoles in Washington Square Park

Male home ranges varied from 18.15 m^2^ to 846.21 m^2^, with a mean male home range size of 260m^2^. Female home ranges varied from 16.06 m^2^ to 1,537.67 m^2^, yielding a mean female home range size of 410 m^2^. A general linear model revealed that the home range size of males (*n* = 42) was smaller than it was for females although these differences were only marginally significant (*n* = 39) (*F*_1,9_ = 4.107, *p* = 0.046) (Figure 2).

### 3.2 Relationship between home range size and body size

There was no relationship between overall home range size and SVL when males and females were combined (Pearson's correlation = −0.107, *p* = 0.580), and when only females (Pearson's correlation = −0.107, *p* = 0.511) or males (Pearson's correlation = −0.107, *p* = 0.361) were considered.

### 3.3 Spatial distribution of males

**FIGURE 3:**
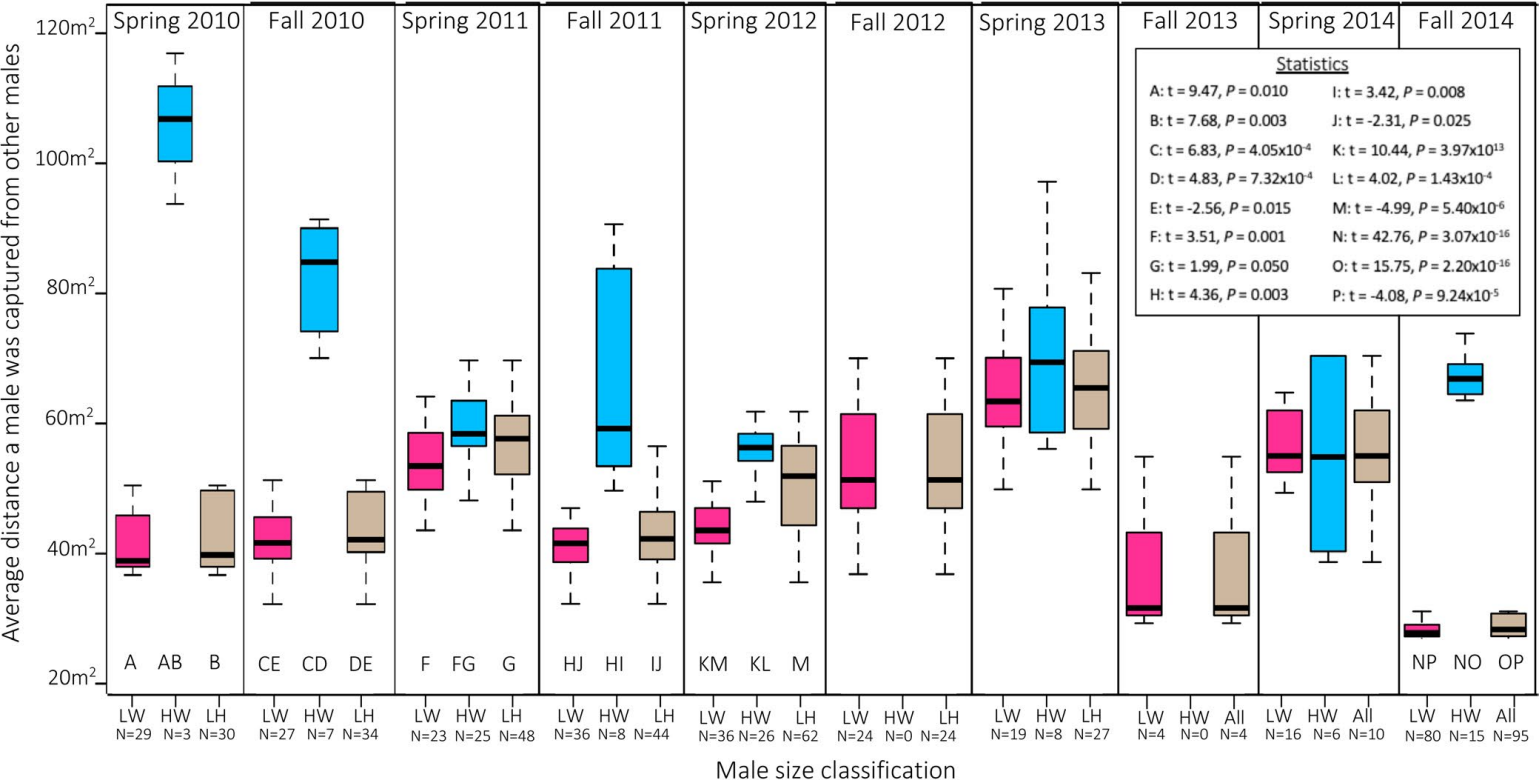
Average distance males maintain from other males. Acronyms for male size classification are as follows: LW = average distance lightweight males distance themselves from each other, depicted with pink boxplots, HW = average distance heavyweight males distance themselves from each other, depicted with blue boxplots, All = average distance all lightweight males distance themselves from heavyweight males, depicted by tan boxplots. Letters below boxplots indicate significant differences with other size classifications; those statistics are reported in the upper right insert

Across all years, the average geographic distance between lightweight males, (*n* = 285) was 69.7 m ± 65.6 (*df* = 284), the average distance between heavyweight males (*n* = 105) was 70.7 m ± 45.7 (*df* = 103), and the average distance between a lightweight and a heavyweight males (*n* = 388) was 50.1 m ± 17.3 (*df* = 287). General linear models revealed that both body size (*p* < 0.001) and cohort (*p* < 0.001) influenced the pattern of spatial distancing in the whole dataset (LM; *r* ^2^ = 0.334, *F*_3,742_ = 125.6, *p* < 0.001). When each cohort was evaluated independently, heavyweight males maintained greater distances between each other than lightweights did from other lightweights in Spring and Fall 2010; in the Spring and Fall of 2011; the Spring of 2012; and in Fall 2014. In all seasons except for spring 2012, lightweights were not significantly more spaced from heavyweights than they were from other lightweights (see Figure 3). We conducted partial *F* tests using a chi‐square distribution that showed inclusion of male density in these models, did not change or improve them, so it was not used as a covariant in the final analysis. This means that within this population, heavyweight males spaced themselves more apart than do lightweights, but lightweights do not space themselves out farther from heavyweights than they did from other lightweights.

**FIGURE 4:**
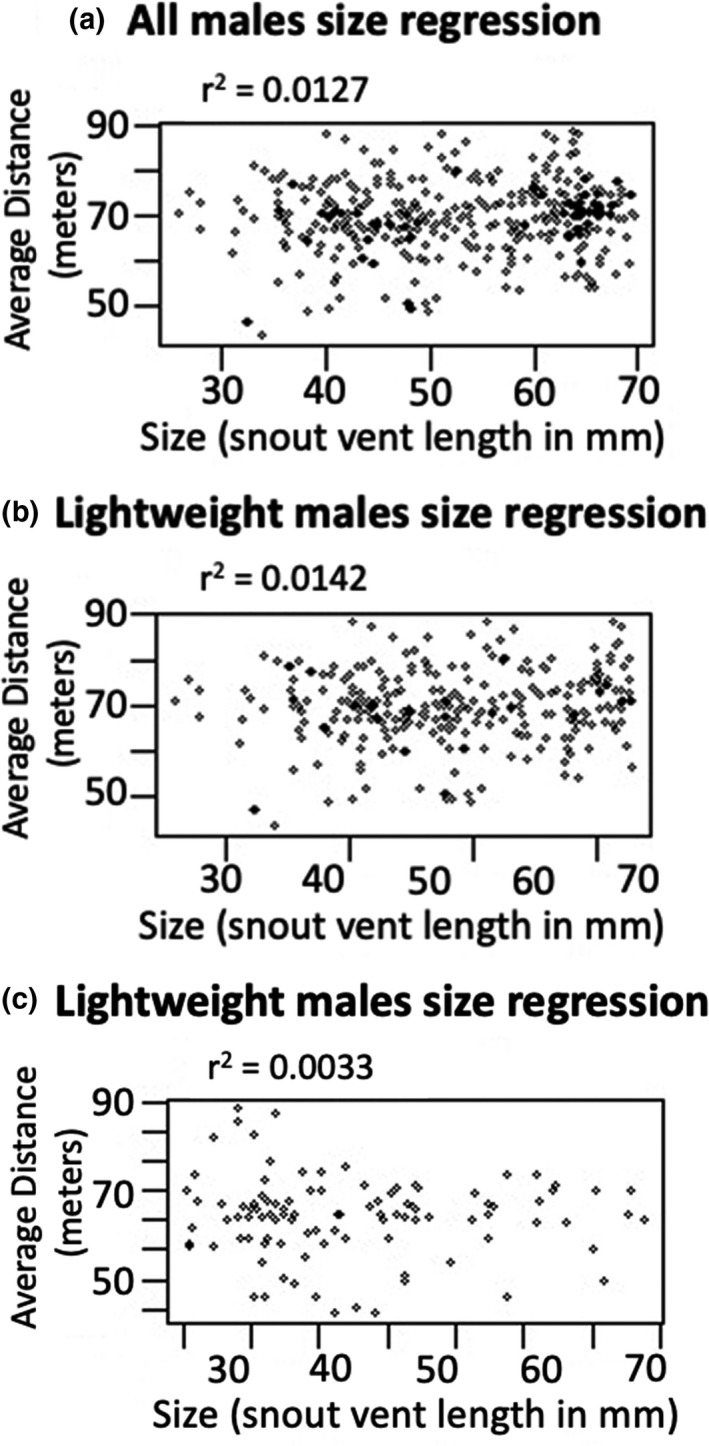
The relationship between average distance and differences in body size. (a) Includes all males. (b) Lightweights only. (c) Heavyweights only

**FIGURE 5:**
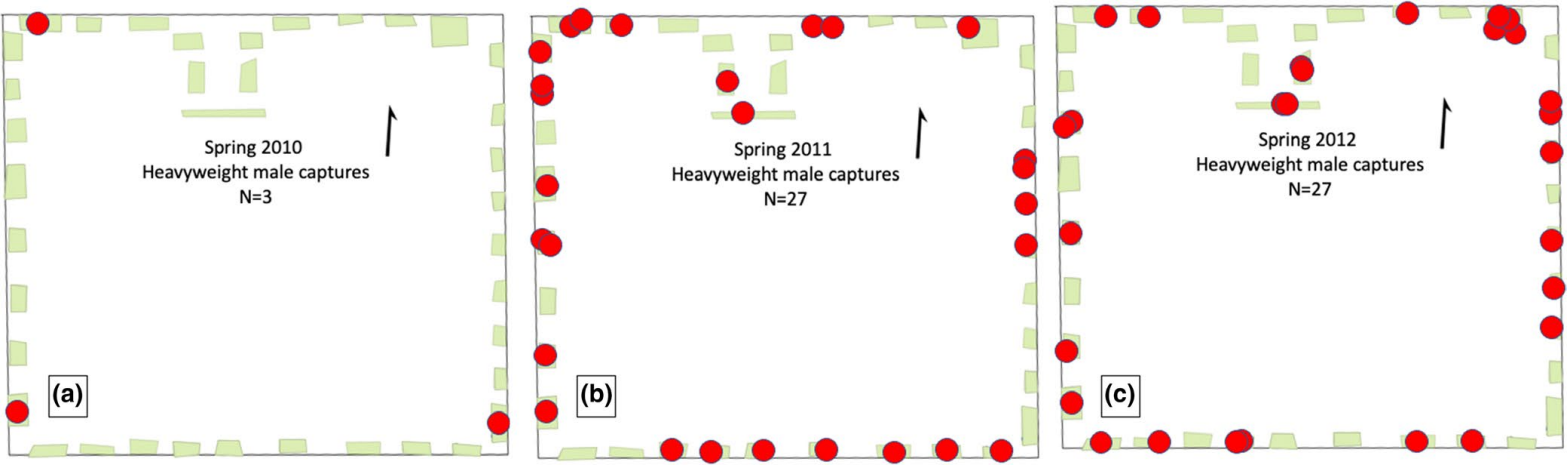
Examples of male spatial distributions, with emphasis on heavyweight males. In the representation, red dots represent an individual heavyweight male. Every capture season can be seen in supplemental materials

Across all cohorts, Mantel tests showed that there was no evidence for a significant relationship between differences in male size and geographic distance between males (*N* = 389, *R* ^2^ = 0.0127, *p* = 0.083). This relationship was not significant when considering only heavyweights (*N* = 105, *R* ^2^ = 0.0033, *p* = 0.138) or lightweights (*N* = 285, *R* ^2^ = 0.0142, *p* = 0.109) (Figure 4). However, examination of QGIS spatial maps across successive years supported the finding that heavyweights may space themselves out more than lightweights in cohorts with low population densities. When the number of heavyweights was low (*n* = 3/9.2 km^2^), as it was in the Spring of 2010, heavyweights were captured at the maximum possible distance from one another (Figure 5a). When the number of heavyweights was high (*n* = 27/9.2 km^2^), distances between them were low and usually one heavyweight was found per bush (Figure 5b), although occasionally two or more heavyweights were captured in the same bush. Since our sampling essentially represents snapshots of individual locations at any given sampling time, this may indicate incursions by neighboring heavyweights (Figure 5c).

### 3.4 Impact of relatedness on the spatial distribution of males and females

Genotyping produced unique microsatellite genotypes for 846 individuals. ACAR 19 was the only locus to not show deviations from Hardy–Weinberg equilibrium (skewed toward homozygosity), and there were no null alleles detected in the data set. We also observed 10 instances of linkage disequilibrium but no consistent pattern between loci (Table S2). The number of alleles per locus ranged from 13 to 33 with a mean of 19.14. The average observed heterozygosity was 0.6381 ± 0.2455, and the expected 0.7286 ± 0.2326.

Across all cohorts, relatedness assessment by SPAGeDi revealed that a heavyweight is on average less related to its neighbors (coefficient of relatedness [*k*] = −1.44 × 10^–2^ ± 3.38 × 10^–4^) than it is to the rest of the male population (k = 1.50 × 10^–3^ ± 3.18 × 10^–3^) (*t* = 0.106, 121*df*, *p* = 0.008). When other neighboring heavyweight males were excluded from this same analysis, there was no difference between the relatedness of a heavyweight and its neighbor (which would be a lightweight) versus the rest of the male population (*F* = 1.13_10,121_, *p* = 0.366).

Spatial auto‐correlograms revealed an inconsistent pattern of IBD across cohorts. In females, a significant pattern of IBD was observed in only six cohorts (Spring 2010, Fall 2010, Spring 2011, Spring 2013, Spring 2014 & Fall 2014), but in those cohorts there was no consistent distance class in which IBD was detected each time (see Table S4). Additionally, a general Mantel test over all distance classes in each cohort showed no significant IBD relationship (*r* ^2^ = −0.005, *p* = 0.487). A nonsignificant pattern of IBD was also observed within all male cohorts (*r* ^2^ = −0.002, *p* = 0.510) (Table 1) (see supplemental material for all auto‐correlograms, Figure S2).

**TABLE 1:** Explanation of statistical test

Statistic	Associated hypothesis	Software	Method
Home range size as a consequence of sex	1	R	General linear model
Home range size as a consequence of body size	1	R	Pearson's correlation
Average distance of males from all others (whole dataset)	2	R	General linear model
Average distance of males from all others (within cohort)	2	R	Wilcox test
Pedigree Construction	3	COLONY	Sequential cohort addition
Coefficient of relatedness between males	3	SPAGeDi	Pairwise relatedness values
Isolation by distance in males and females	3	R	Mantel test
Pairwise relatedness between males as a consequence of average distance	3	R	General linear model
Pairwise relatedness as a consequence of body size	3	R	General linear model
Offspring territory inheritance	4	R	Pearson's chi‐square

### 3.5 Home range inheritance

**FIGURE 6:**
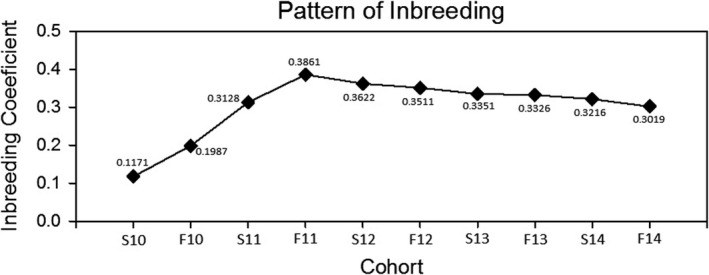
The pattern of inbreeding in the Washington Square Park population of *Anolis carolinensis* over the sampling period. S = a spring collection period, F = a fall collection period, and the year follows each seasonal indicator

Following pedigree construction (see Table S3 for constructed pedigree), sufficient parentage and geographic information was available in our dataset to evaluate 16 sires and 10 dams for offspring home range inheritance in 65 offspring. Results indicate that 35% of female offspring and 36% of male offspring were found within their mother's home range. A chi‐square test (*x* ^2^ = 0.707) suggested this relationship is no different than what would be expected from a random sampling (Table S3). By contrast, 50% of female offspring and none of the male offspring were found within their father's home range; here, a chi‐square test (*x* ^2^ < 0.01) suggested the relationship was not at random (Table S3). We also observed that the inbreeding coefficient (*F*) of the population gradually increased with time and sampling, stabilizing at 0.338 ± 0.0258 after 10 cohorts (Figure 6).

## 4 DISCUSSION

The way individuals arrange themselves relative to each other within the environment is a fundamental property of population ecology that influences dispersal, mating system, and individual fitness. However, the genetic and social factors underlying spatial distributions of individuals within a given habitat are potentially complex and poorly understood for most animal species. Here, we used a combination of body size, locality, and relatedness measures to test four hypotheses and associated predictions regarding the factors influencing spatial distribution of free‐ranging green anoles in an urban habitat.

Our first hypothesis (that home range size is greater in males than females) was not supported. Although males tend to maintain larger home ranges in most animal species (Cederlund & Sand, 1994), we found that female green anoles in our study population maintain home ranges more than 1.5 times greater on average than those of males (Figure 2). However, this difference was only very marginally significant (*p* = 0.05). Studies of sex differences in *Anolis* species home range or territory size are sparse but tend to show that males maintain both larger home ranges (e.g., *Anolis lineatopus* [Rand, 1967]) and larger territories (*Anolis cristatellus* and *Anolis acutus*; [Philibosian, 1975]) than females. With regard to *A. carolinensis*, Jenssen and Nunez (1998) found that green anole males in Georgia maintained home ranges on average eight times larger than those of females, a result which contrasts sharply with ours. However, although Schoener and Schoener (1982) reported male‐biased sex differences in home range for four species of Bahamian anoles, they also demonstrated marked intraspecific variation in those sex differences across multiple sites, potentially driven by variation in both body size and population density. Jenssen et al., (1995) established males maintained a territory on average of 173.6 m^3^; however, we were unable to rigorously assess territory size here because animals were deliberately not captured multiple times within a season. The reason for the differences in home ranging between the Washington Square Park population and other populations is unclear. It could be driven by differences in the type and quality of habitat, which can be substantial (e.g., [Edwards & Lailvaux, 2012; Irschick et al., 2005]) or by general differences between the urban nature of the Washington Square Park population and the rural populations studied by Jenssen and others (Lailvaux, 2020).

Our second hypothesis (home range size is correlated with body size) was also not supported by the data. In our data set as a whole (including both males and females), we found no significant relationship between body size and home range area. In this population, larger individuals do not appear to maintain larger home ranges. This finding is contradictory to other research in lizards. For instance, a previous study on the side blotched lizard (Christian & Waldschmidt, 1984) found a positive correlation between body size and home range area, in both male and females. This association has also been demonstrated in 12 other terrestrial lizard species (Turner et al., 1969). In their review, Christian and Waldschmidt (1984) discuss an additional 16 lizard species, 10 if which are insectivores like *A. carolinensis*, that exhibit the same pattern and suggest that larger lizards with greater metabolic demands require a larger home range. The lack of a relationship between body size and home range size in this study may be due in part to the fact that individuals have a clear preference for cast‐iron plant bushes and avoid excursions into the urban surroundings (see also Irschick et al., 2005), thereby limiting foraging opportunities.

Our third hypothesis (male–male spatial distribution is positively correlated with differences in body size) was partially supported. There is ample empirical evidence that the green anole mating system is dominated by male territoriality and combat (Jenssen et al., 2000, 2001; McMann, 1993; Stamps, 1983; Stamps & Krishnan, 1997 & 1998); consequently, in this case, lightweight males might opt to position themselves as far from heavyweights as possible to avoid injury causing encounters (Irschick & Lailvaux, 2006; Lailvaux et al., 2004). However, if this were the case, then the average distance between individuals should increase with differences in individual size since smaller males will avoid larger ones. Our data do not support this scenario; instead, we found that only certain heavyweight males maintained the greatest distances from each other, and the generally weak relationship between differences in male size and average distance between individuals suggest that male green anoles do not base their proximity to other males based on size alone. However, an important insight into the documented *A. carolinensis* male dimorphism contributed by these data is that the density of heavyweight males in this population is dynamic, and thus, the influence of heavyweight abundance on the spatial distribution of individual animals (if any) might be both variable and transient.

Because of both the size dimorphism in adult male green anoles and the likelihood of male offspring dispersal, we expected to see very little pattern of relatedness between lightweight males and the heavyweight males nearest them. As lightweight males mature, they most likely take on the “sneaker male,” running in and out of territories sneaking copulations while avoiding territorial heavyweights (Orrell & Jenssen, 2003). At this point, relationship structure should break down as a result of unrelated lightweight male incursions (Massot et al., 1992). This expectation was only partially supported; heavyweights were no more related to neighboring individuals than the rest of the population. However, neighboring heavyweights were less related to the *each other* than to the rest of the population, suggesting that closely related heavyweights are not inhabiting territories near to each other. Although relatedness is an important aspect of population structure and ecology in many other terrestrial vertebrates (e.g. Vangestel et al., 2011; Zedrosser et al., 2007]), this does not appear to be the case in lizards (but see Ryberg et al., 2004). Our result that neighboring heavyweights tend to be unrelated is unlikely to be a result of deliberate kin avoidance either and is probably a consequence of male‐biased dispersal (see below).

Territory locations, as well as the identity of the holder, appear to be initially dictated by winter behavior in the green anole (Jenssen et al., 1996). After overwintering in leaf litter (and opportunistically basking), females may emerge and remain in the same area if the resources are sufficient or range further if not. Males, on the other hand, will emerge and evaluate areas for the presence of females (Jenssen et al., 2001). This, too, may be the same location as a wintering habitat; however, the dynamic of male size could dictate at this point whether an emerging male will claim that territory, or be forced out by a stronger male (Wiggett & Boag, 1992). In our dataset, a given heavyweight male appears to actively defend a single bush, which is inhabited by unrelated lightweight males and multiple females, including daughters (although we again note that we could not rigorously estimate territory size; see above). The absence of male offspring within a bush of a father may be an indication that the male offspring are being forced out of their father's territory (Charnov & Berrogan, 1993; Kopp et al., 2015). A nomadic strategy may therefore be best for lightweights in this population roaming through one territory into another and sneaking copulations with females until they are large enough to attain heavyweight status and hold territories of their own (Orrell & Jenssen, 2002, 2003; Sinervo & Lively, 1996).

Our final prediction (females will inherit territories from their mothers, but males will not inherit territories from their fathers because of male‐biased dispersal) was not supported by our data. Only 35% of daughters and 36% of sons were found in their mother's territory, meaning that almost two thirds of them dispersed or died. This result is consistent with studies of maternal relatedness and social structure in some other lizard species; for example, Qi et al., (2012) showed that juvenile burrowing sand lizards do not preferentially share burrows with their parents. However, our data go further than this; on no occasion was a male offspring found in the territory of its father, yet 50% of a father's daughters were found within his territory. These findings indicate that the pattern seen in father/offspring space use was not random, while mother/offspring space use may have been. The absence of male offspring within his father's bush may be an indication that the male offspring are being forced out of their father's territory (Charnov & Berrogan, 1993; Kopp et al., 2015).

The elevated inbreeding coefficient from this analysis also suggests that there is the potential for as much as two generations of first order inbreeding between the parents and offspring in this population. Female offspring hatch and seem to remain in their natal home range, only as long as resources permit (Wiggett & Boag, 1992). As females get older, they then disperse, most likely the result of population density increases, and avoidance of kin competition within the natal bush (La Galliard et al., 2003; Olsson & Shine, 2003). However, the female offspring that remain could be the offspring of that bush's occupying heavyweight, and on occasion it appears likely that some reproductive females may be mating with their fathers. Inbreeding tolerance has been observed not only in lizard species (Richard et al., 2009), but also in aquatic (Neff, 2004) and avian taxa as well (Bateson, 1982; Cohen & Dearnorn, 2004).

In conclusion, we have shown that relatedness has no effect on the distribution of individuals within a population of green anoles in Washington Square Park and that body size appears to influence the spatial distribution of heavyweight males, albeit more so at low population densities. The data showing that neighboring heavyweights are less related to one another, likely due to the impacts of population density and male offspring dispersal, not relatedness. Our data also support a pattern of male‐biased dispersal in green anoles, but because IBD in female cohorts is inconsistent this data does not provide convincing evidence for philopatry in females. However, female presence in the home ranges of their fathers might support this idea with additional investigation. These results shed light on the factors driving home range establishment in a model organism for ecology and evolution and illustrate the value of integrating complementary approaches to understanding the population ecology of cryptic species.

## CONFLICT OF INTEREST

None declared.

## AUTHOR CONTRIBUTIONS

**W. David Weber:** Conceptualization (lead); data curation (lead); formal analysis (lead); investigation (lead); methodology (lead); project administration (lead); supervision (lead); validation (lead); visualization (lead); writing – original draft (lead); writing – review and editing (lead). **Nicola M. Anthony:** Conceptualization (equal); formal analysis (supporting); funding acquisition (supporting); methodology (equal); resources (supporting); software (equal); supervision (supporting); validation (equal); writing – original draft (equal); writing – review and editing (equal). **Simon P. Lailvaux:** Conceptualization (equal); data curation (equal); formal analysis (supporting); funding acquisition (lead); investigation (supporting); methodology (equal); project administration (supporting); resources (equal); software (supporting); supervision (supporting); validation (equal); visualization (equal); writing – original draft (equal); writing – review and editing (equal).

### OPEN RESEARCH BADGES

This article has earned an Open Data Badge for making publicly available the digitally‐shareable data necessary to reproduce the reported results. The data is available at https://doi.org/10.5061/dryad.zcrjdfn8w.

## Supporting information

Supplementary Material

## Data Availability

DRYAD (https://datadryad.org/stash/share/aPcTE9_zOHl4zxRlp7NFGsuGEqsf6TEsUGjYbtRUU3o).
