# Supplementary material for: Size but not relatedness drives the spatial distribution of males within an urban population of Anolis carolinensis lizards
Source: Ecol Evol. 2021 Feb 14;11(6):2886–98. doi: 10.1002/ece3.7248 (PMC7981233; doi:10.1002/ece3.7248)

Supplemental Material.

Supplemental table S1; Multiplex PCR primer information, "Exp"=Expected, "Obs"=observed, "(bp)"=base pair

|  | Primer | Exp. Range (bp) | Obs. Range (bp) | Forward Primer (fluorophore label) | Reverse Primer | Repeat Motif |
| --- | --- | --- | --- | --- | --- | --- |
| Multiplex 1 | ACAR1 | 110-122 | 100-110 | CCAAAAACCAAAAAGGCTGA(Blue) | TGGACACACATACACCCACA | (AC)_38_ |
|  | ACAR8 | 129-177 | 150-180 | CCCAATAGAGGAAAGGGACC (Green) | AGAATCACGCCTTCTGCTTT | (AAAG)_76_ |
|  | ACAR19 | 210-234 | 220-260 | GAAAAGTAGTGGGGCATTGG(Yellow) | AGTTTCCCAAGAAAACCCGT | (AG)_42_ |
|  | ACAR30 | 236-283 | 220-280 | CATCTCTTCAGGCTTTTGCC(Blue) | CTGTCTCTTCCTCCACCTGC | (AAAG)_48_ |
| Multiplex 2 | ACAR2 | 99-125 | 100-110 | GAATGAAGCTAAGGGGCACA(Blue) | AGCAGATGGAAGGAAAGCAA | (AC)_28_ |
|  | ACAR9 | 148-196 | 180-200 | AAAGGCAATGGCAGAGAAAA(Green) | TAATGGGAAAGGAGGCAGTG | (AAGG)_52_ |
|  | ACAR10 | 180-198 | 160-180 | GGATGTGTGTGTTTGTGTTGG(Yellow) | GGCTGTTGAGGGATTCTTGA | (ACAT)_28_ |
|  | ACAR36 | 230-253 | 230-310 | TTGCTGCTGCTGATGTCATT(Blue) | TCCCATTAAAAATCATGCTGC | (AAAG)_80_ |

Supplemental table S2; Summary of Linkage Disequilibrium. As linkage disequilibrium was don detected in all capture seasons, we have only included seasons in which it was (left column). In the right column, we have indicated the loci that were in disequilibrium during that season.

| Season | Linkage | | |
| --- | --- | --- | --- |
| Spring 2010 | Acar1 | ↔ | Acar30 |
| Fall 2010 | Acar9 | ↔ | Acar36 |
| Spring 2011 | Acar2 | ↔ | Acar10 |
|  | Acar2 | ↔ | Acar19 |
|  | Acar10 | ↔ | Acar19 |
|  | Acar2 | ↔ | Acar36 |
| Fall 2012 | Acar2 | ↔ | Acar10 |
| Fall 2014 | Acar1 | ↔ | Acar10 |
|  | Acar10 | ↔ | Acar19 |
|  | Acar10 | ↔ | Acar36 |

Supplemental table S3; Colony Pedigree. Listed in this table are all capture captured individuals. Parentage inferred by the program is denoted by an “D” prefix for dams and “S” for sire. Because these individuals were inferred they were excluded from analysis, but included here for transparency. OID=offspring identification, FID=father identification, MID=mother identification.

Supplemental table S4; Spatial correlogram results. With a spatial correlogram it is possible to visualize the relationship between relatedness and geographic distance (see correlograms in supplemental figure S2); however, that visualization must be supported with statistical analysis, namely a Mantel test. Here we have used the symbol "-" to indicate when isolation by distance (IBD) was visualized graphically within a cohort; meaning that on average, individuals of that cohort became less related as geographic distance between individuals increased. We used the symbol "+" the opposite relationship, meaning that on average, individuals of that cohort became more related as geographic distance between individuals increased. We then used the symbol "?" to indicate where no pattern was discernable. In all cohorts (including those where IBD was visualized, Mantel test suggest that no cohort showed a statistically significant pattern)

|  | Spring 2010 | Fall  2010 | Spring 2011 | Fall  2011 | Spring 2012 | Fall  2012 | Spring 2013 | Fall  2013 | Spring 2014 | Fall  2014 |
| --- | --- | --- | --- | --- | --- | --- | --- | --- | --- | --- |
| Female | **-** | **?** | **?** | **-** | **+** | **+** | **?** | **+** | **?** | **?** |
| Male | **+** | **-** | **?** | **-** | **?** | **-** | **?** | **?** | **+** | **?** |

Supplemental figure S1; Cohort capture maps


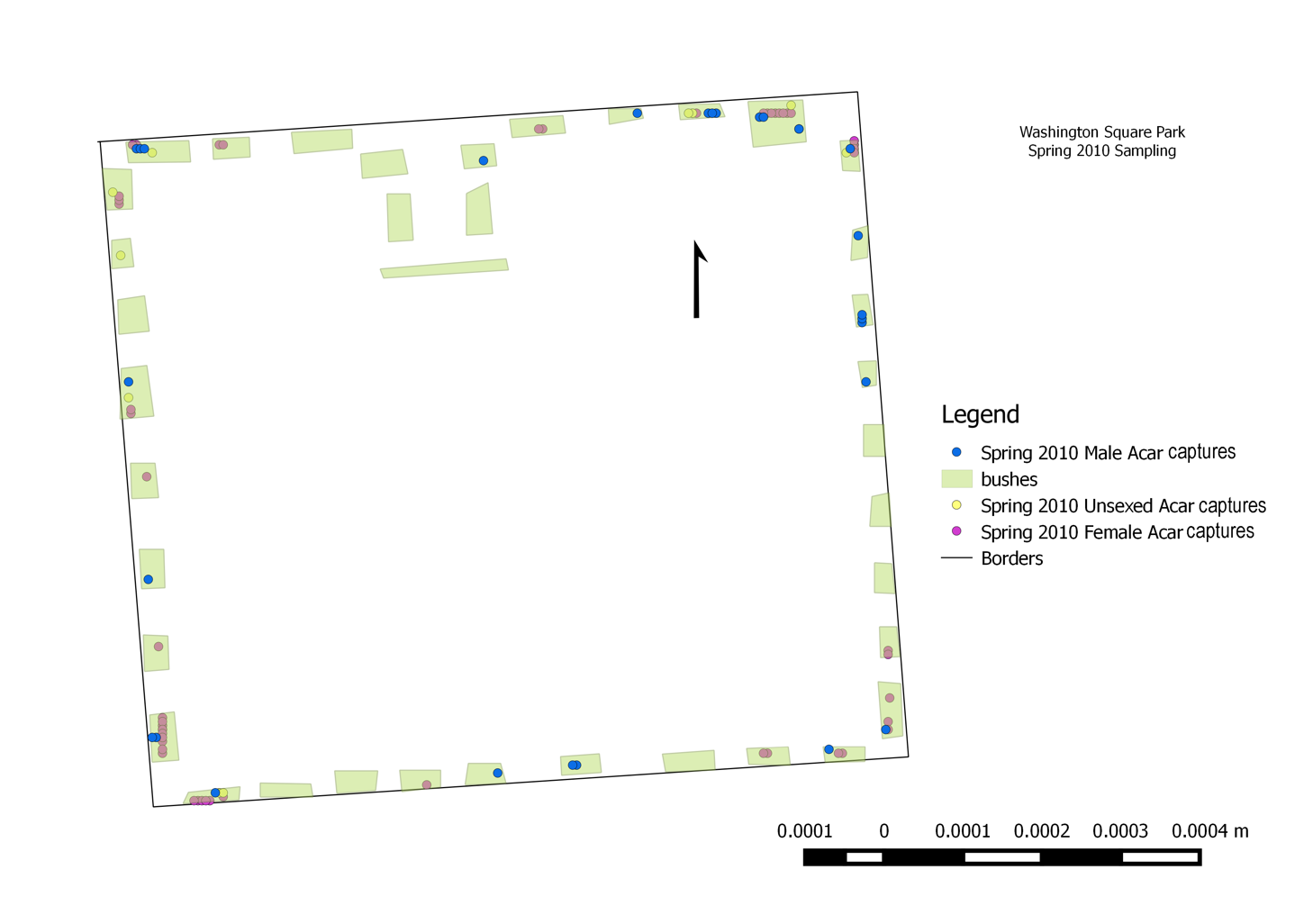


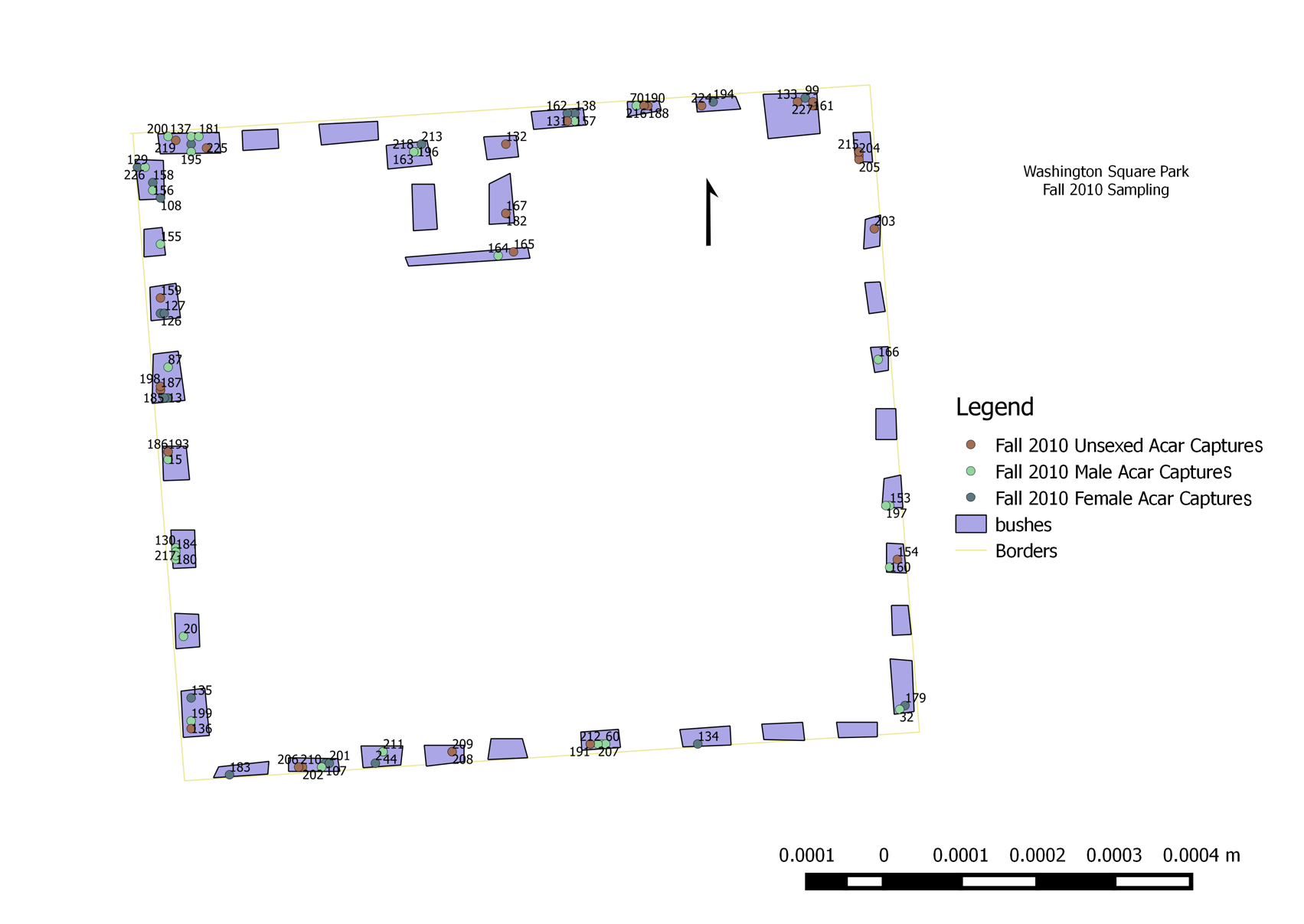


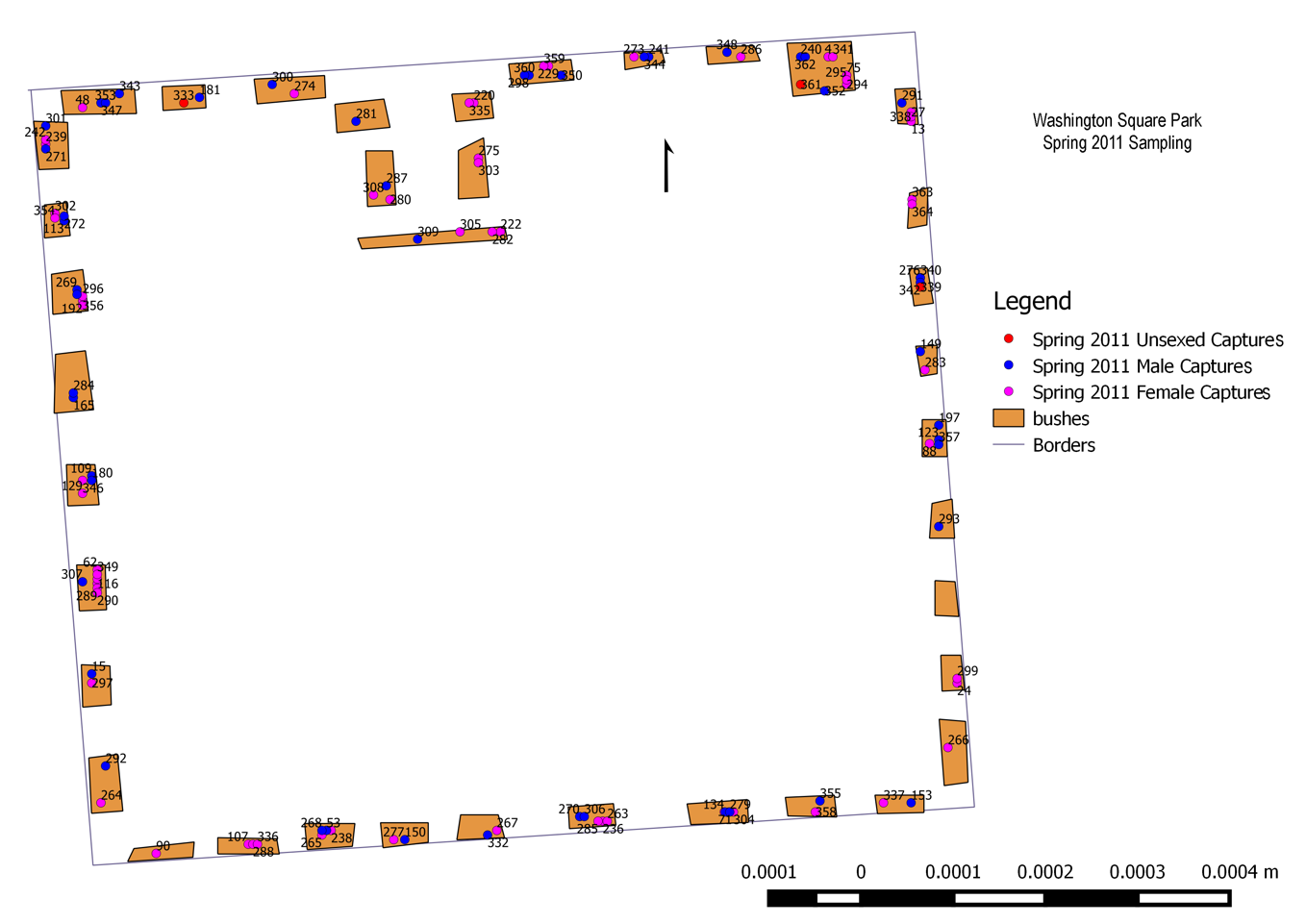


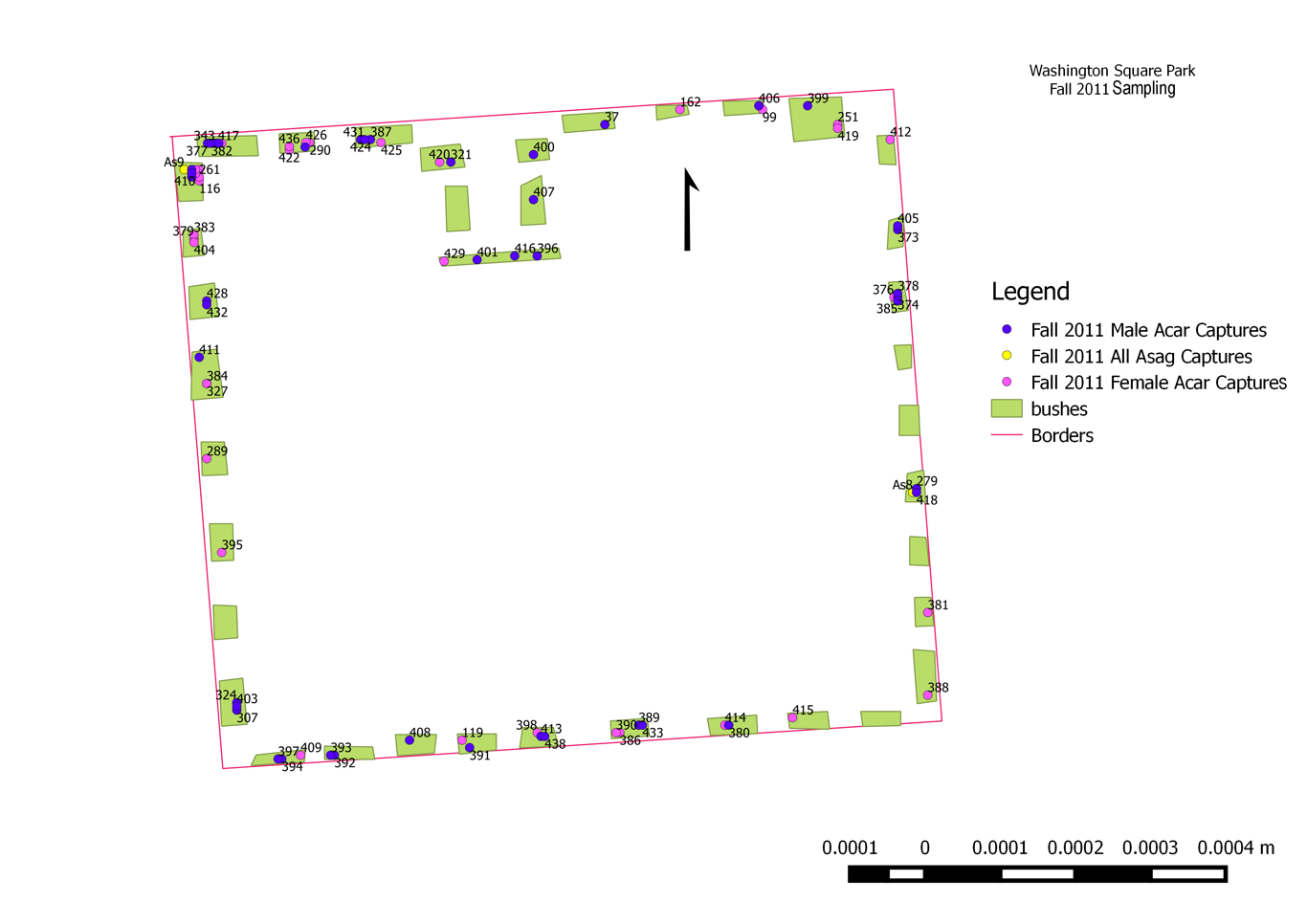


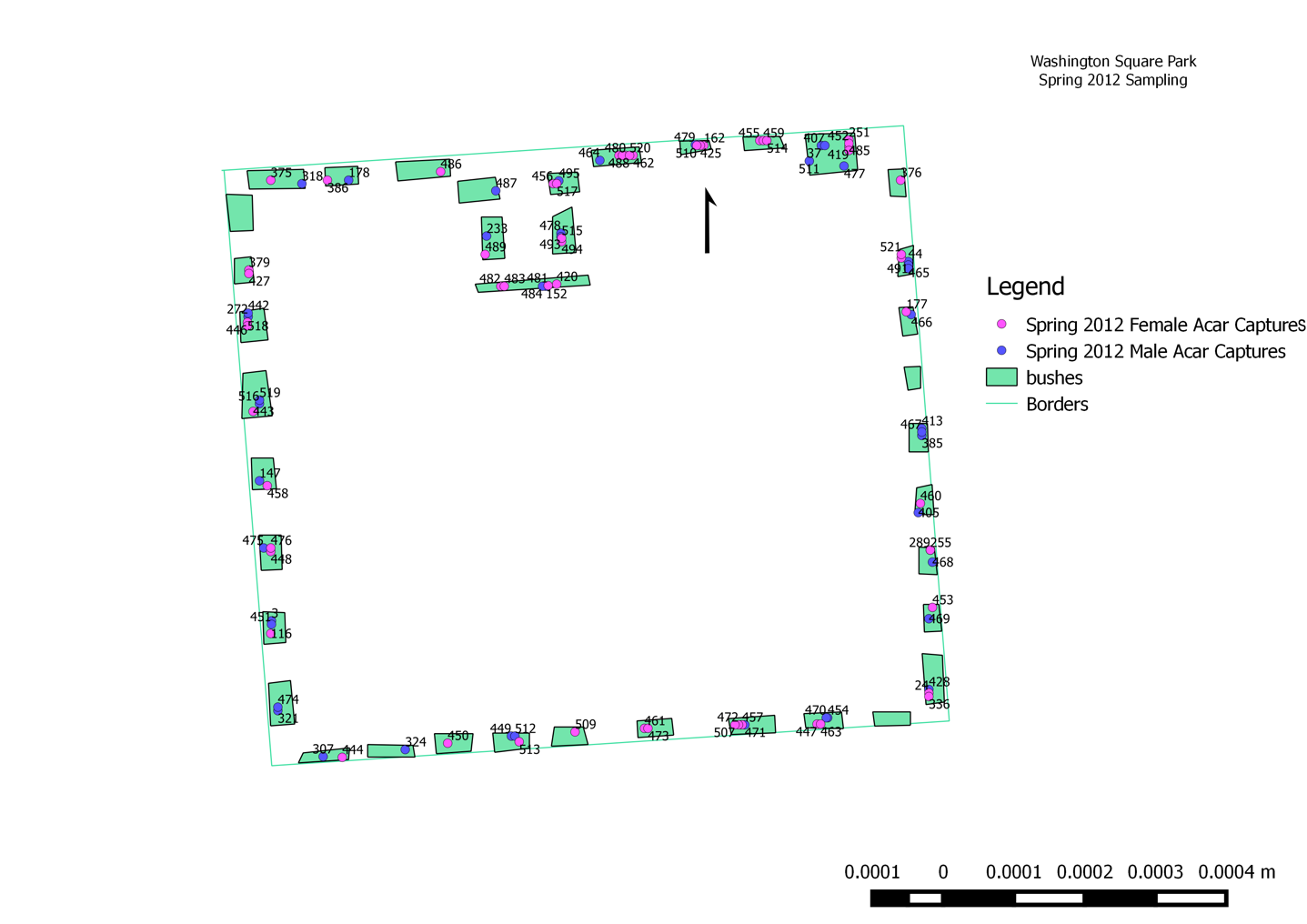


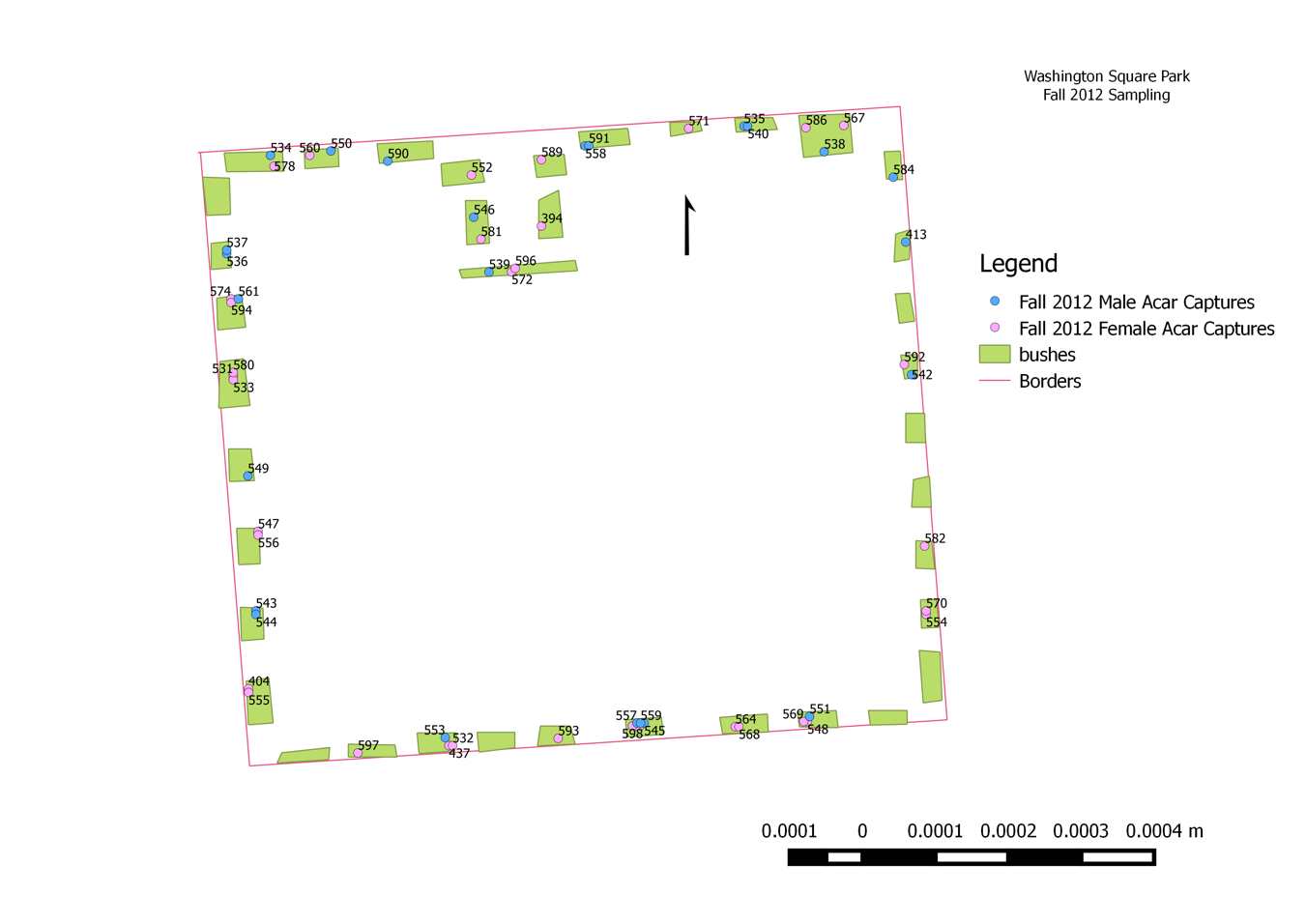

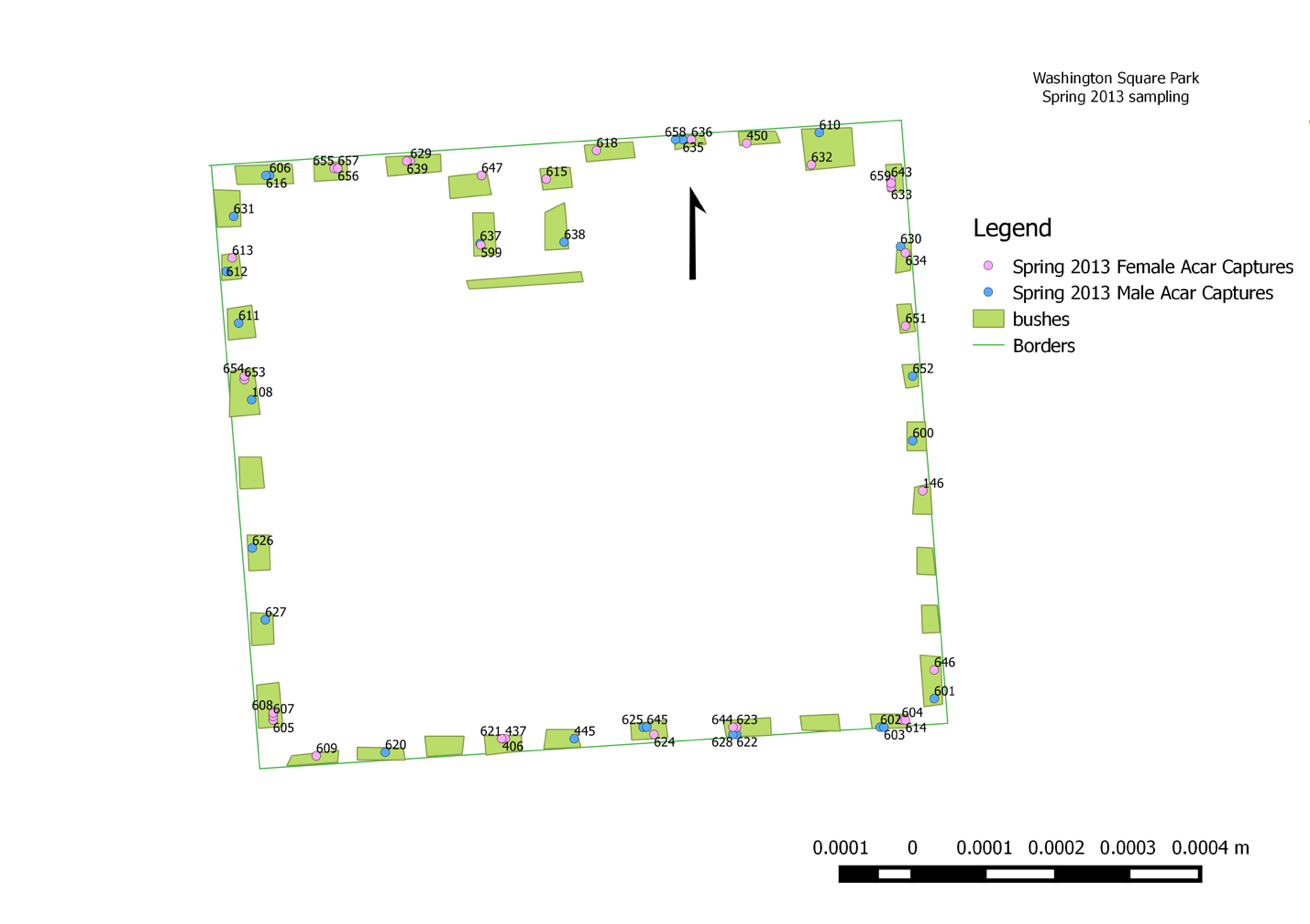


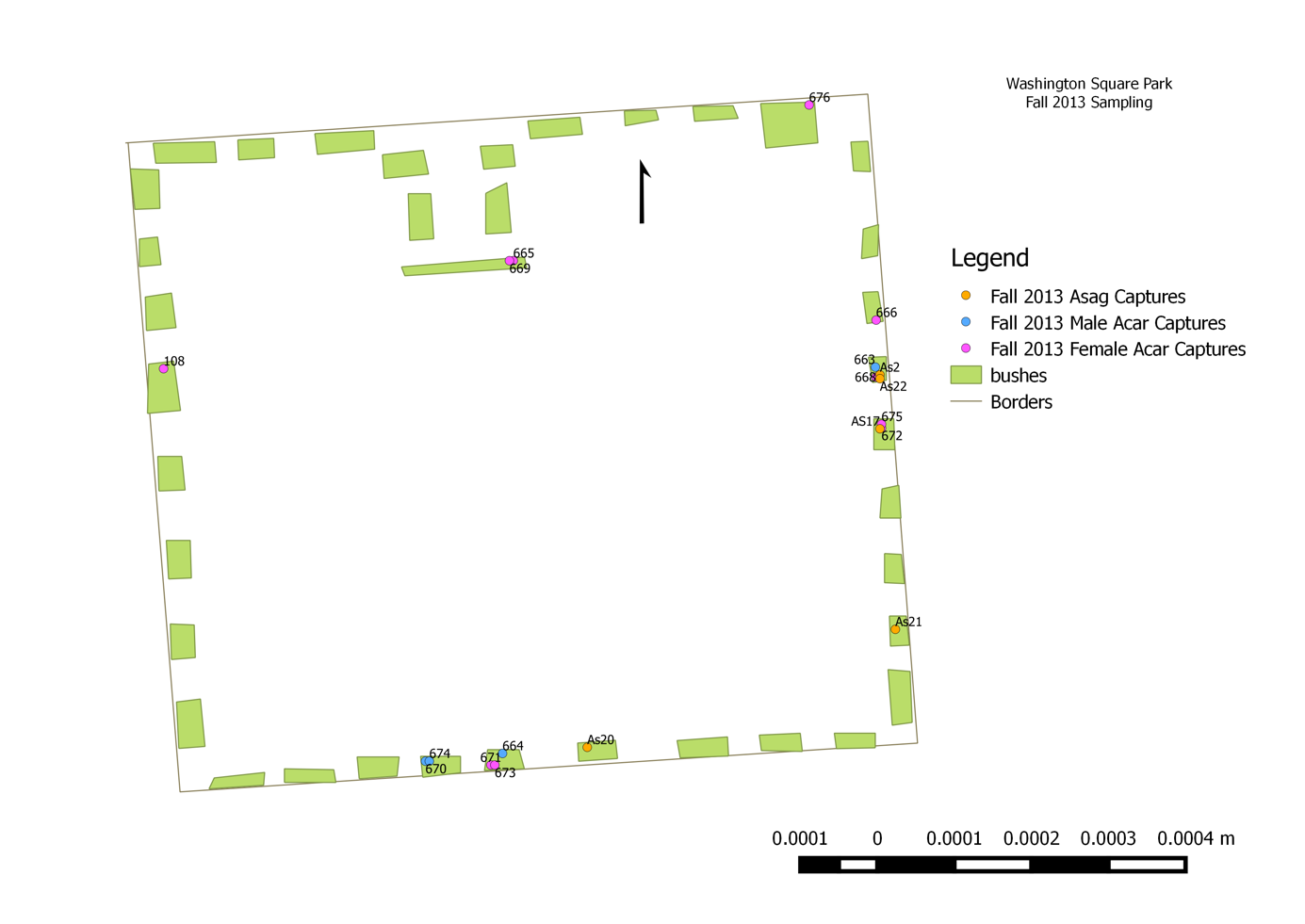


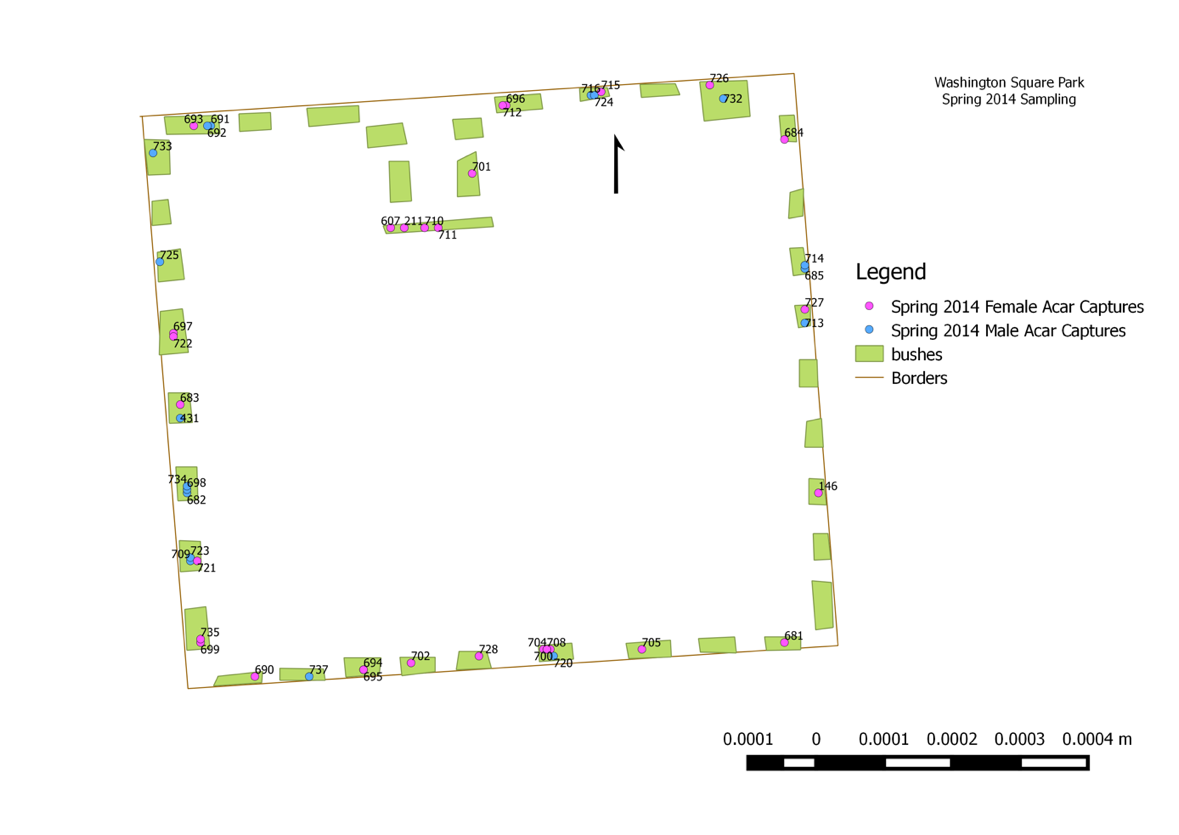


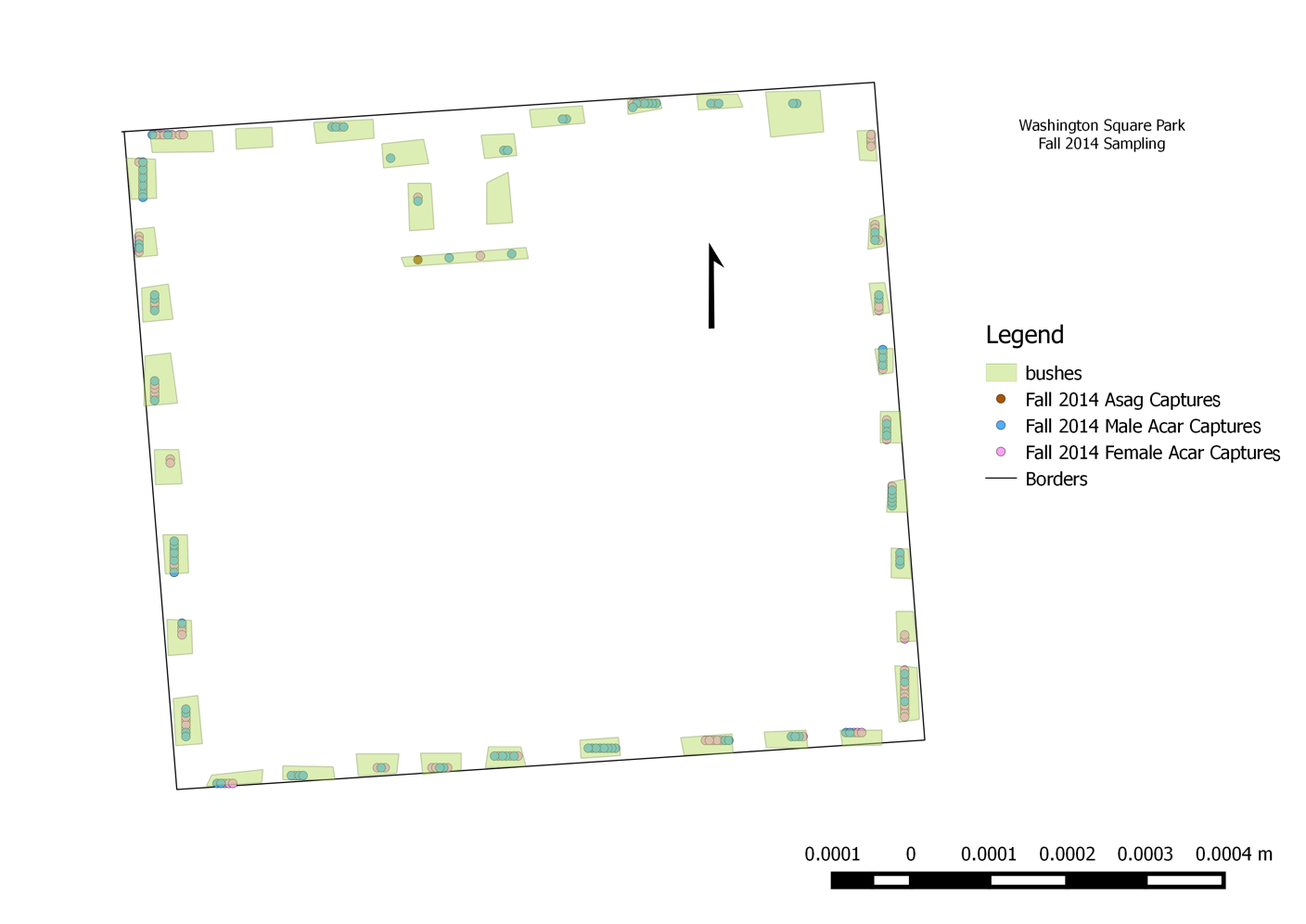


Supplemental figure S2; Spatial correlogram. For each sampling period there are two correlogram, one for male captures and one for female captures. The program SPAGeDi established five distance classes along with kinship coefficients for each cohort. The upper limit of those distance classes is plotted on the x-axis, and kinship coefficient on the y-axis. Plotted on the graph is the average kinship coefficient for each distance class. Dotted lines in the graph represent the 95% confidence intervals. The relatedness trendline and slope indicate the direction of relatedness as desistance increases. When the majority of the data points trend downward and fall within the critical values, there is strong evidence for isolation by distance.

Supplemental table 5: *Anolis segrei,* population in Washington Square Park. At the time of this work the Anole population was predominantly made up of *A. carolinensis*, as we continued through the seasons there were very small numbers of *A. segrei* observed in the park, we captured and documented their data as well, but it was not included in the work, as their numbers were so small, and a single individual *A. segrei* was never recaptured.

| Capture season | *A. Segrei* captured | *A. carolinensis* captured | *A. segrei* percentage of population |
| --- | --- | --- | --- |
| Spring 2010 | 0 | 106 | 0% |
| Fall 2010 | 6 | 143 | 4% |
| Spring 2011 | 0 | 181 | 0% |
| Fall 2011 | 3 | 86 | 3.5% |
| Spring 2012 | 1 | 123 | >1% |
| Fall 2012 | 3 | 74 | 4% |
| Spring 2013 | 3 | 83 | 3.6% |
| Fall 2013 | 4 | 26 | 15% |
| Spring 2014 | 0 | 184 | 0% |
| Fall 2014 | 3 | 183 | 1.6% |

Supplemental Image S1: Washington Square Park. This image in the left panel was taken from the south east corner of Washington Square Park. In the image is the pedestrian sidewalk and rod-iron fencing that encircles the entire park. Just park-side is located an *aspidistra* (cast iron) bush. The bush in this image corresponds to habitat #21 in the right panel.


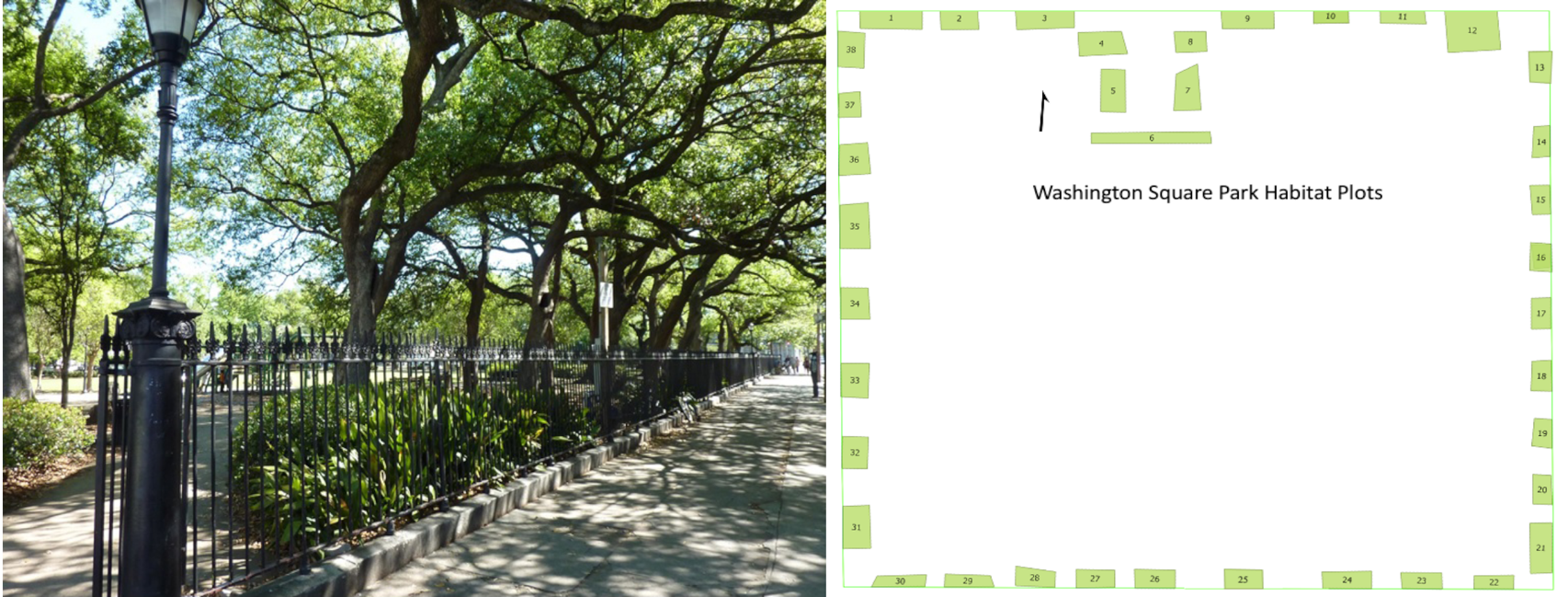


Supplemental figure S3: Polygonal area assessment. The panel on the right depict the capture locations of Individual #13 in the Spring and Fall of 2010, and the spring of 2011. The panel on the left depicts the polygonal area measurement for this individual. That area is recorded as the individual’s home range.


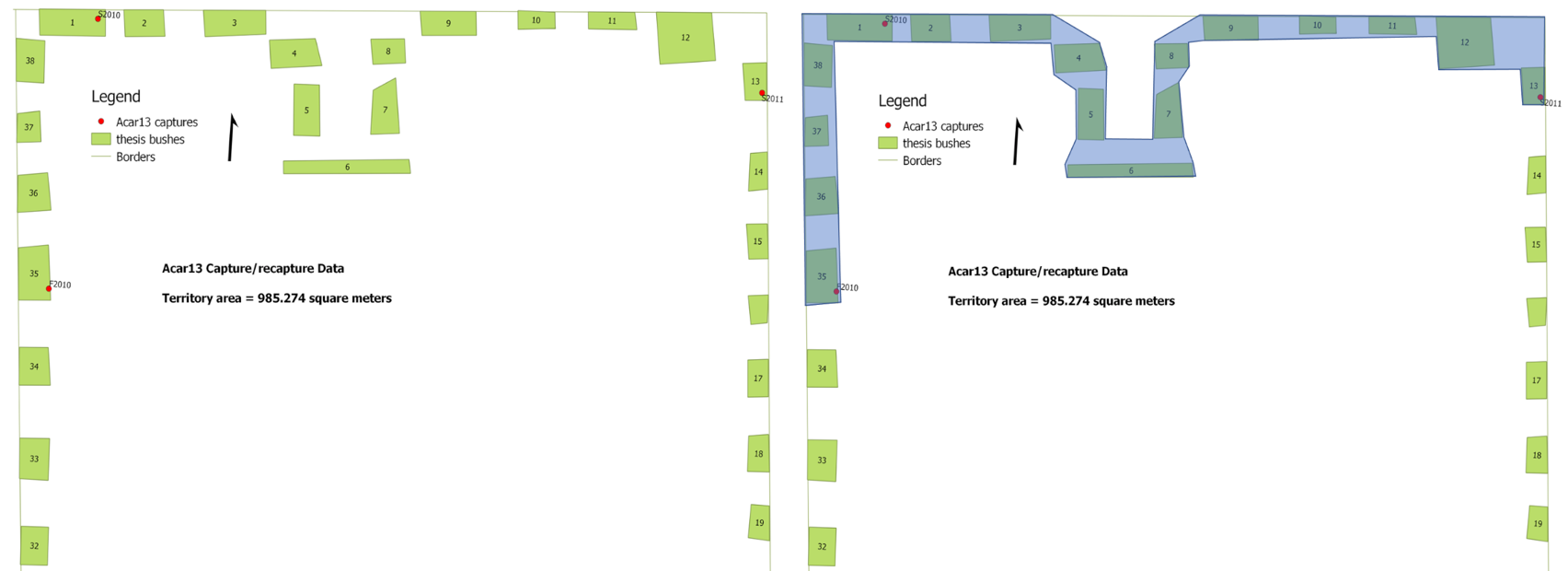

Supplement: Supplementary file 1 — Supplementary Material [file ECE3-11-2886-s001.docx]
